# The Virtual Mouse Brain: A Computational Neuroinformatics Platform to Study Whole Mouse Brain Dynamics

**DOI:** 10.1523/ENEURO.0111-17.2017

**Published:** 2017-06-28

**Authors:** Francesca Melozzi, Marmaduke M. Woodman, Viktor K. Jirsa, Christophe Bernard

**Affiliations:** Aix Marseille Univ, INSERM, INS, Inst Neurosci Syst, Marseille, France

**Keywords:** connectome, fMRI, modeling, resting state

## Abstract

Connectome-based modeling of large-scale brain network dynamics enables causal *in silico* interrogation of the brain’s structure-function relationship, necessitating the close integration of diverse neuroinformatics fields. Here we extend the open-source simulation software The Virtual Brain (TVB) to whole mouse brain network modeling based on individual diffusion magnetic resonance imaging (dMRI)-based or tracer-based detailed mouse connectomes. We provide practical examples on how to use The Virtual Mouse Brain (TVMB) to simulate brain activity, such as seizure propagation and the switching behavior of the resting state dynamics in health and disease. TVMB enables theoretically driven experimental planning and ways to test predictions in the numerous strains of mice available to study brain function in normal and pathological conditions.

## Visual Abstract

**Figure:**
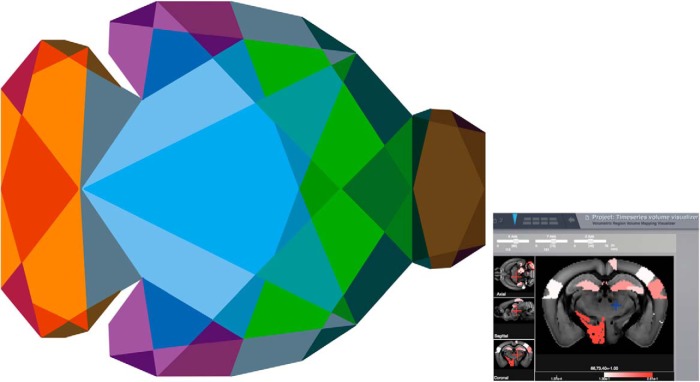

## Significance Statement

The Virtual Mouse Brain (TVMB) represents a versatile and intuitive tool for virtualizing and simulating mouse whole-brain dynamics using a connectome-based model approach. TVMB enables the construction of individual mouse brains using diffusion magnetic resonance imaging (dMRI) experiments. TVMB also allows building detailed connectomes based on tracer experiments realized by the Allen Institute. Various modalities can be modeled [e.g., electroencephalography (EEG) and fMRI]. The platform can be used to generate predictions that can be tested experimentally.

## Introduction

Dedicated software environments are available to simulate detailed neuronal dynamics such as Neuron, Genesis, and MOOSE, which model the complex dendrite geometry, reaction-diffusion processes and receptor distributions of individual neurons and smaller networks (Hines and Carnevale, 1997). To simulate larger networks, neuron models are reduced to point neurons (Izhikevich, 2003; Brette and Gerstner, 2005), which is the case of the simulator BRIAN (Goodman and Brette, 2009), NEST (Diesmann and Gewaltig, 2001), and NENGO (Eliasmith et al., 2012). However, scaling up for detailed models beyond an entire cortical column (Markram, 2012) and a few brain regions becomes quickly intractable even for networks of point neurons. Although neuromorphic computation offers interesting alternatives for the future (the SpiNNaker project http://apt.cs.manchester.ac.uk/projects/SpiNNaker/ and the BrainScaleS project http://brainscales.kip.uni-heidelberg.de/), macroscopic modeling using neural population approaches is the only viable whole-brain network modeling strategy nowadays.

The Virtual Brain (TVB) is an open-source simulation software designed to model whole-brain network dynamics, where the network’s connectivity is based on diffusion magnetic resonance imaging (dMRI)-based individual connectomes or adaptations of more precise primate connectomes (Sanz Leon et al., 2013). TVB comprises several generative neural population models, defined in physical 3D space and constrained by anatomy, allowing simulating neuroimaging signals [such as magneto- and electroencephalography (MEG, EEG), or functional MRI (fMRI)]. Whole-brain dynamics can also be manipulated in TVB, e.g., via stimulation. TVB provides a large set of tools for visualization and data analysis (Sanz-Leon et al., 2015). As such, TVB provides a conceptual framework to interpret neuroimaging data, offering promising diagnostic and therapeutic perspectives (Jirsa et al., 2017; Proix et al., 2017). Other groups demonstrate converging results using similar large-scale brain modeling approaches (Hutchison et al., 2013; Sinha et al., 2017). However, very few model predictions can be experimentally tested in humans for obvious ethical reasons. Thus, assessing causality and extracting general principles of brain dynamics in health and disease remains a challenge.

Rodent research enabled major advances in our understanding of brain function and dysfunction, but mostly at the microscopic scale. The advent of new generations of MRI machines now gives access to detailed anatomic, structural and functional information at the whole rodent brain scale (Stafford et al., 2014), thus providing a formidable opportunity to explore general principles of whole-brain dynamics. Indeed, hypotheses can be tested and causality can be assessed in the numerous transgenic mouse lines that have been generated to study neurologic disorders and to manipulate neuronal networks (e.g., with optogenetics and pharmacomogenetics). However, a conceptual framework is needed to interpret neuroimaging data and generate testable hypotheses. Such framework would considerably accelerate our understanding of the mechanisms controlling and affecting whole-brain dynamics.

Here, we present The Virtual Mouse Brain (TVMB), the first connectome-based simulation platform to study large-scale mouse brain dynamics.

TVMB is integrated into TVB and adapted to the mouse brain to enable its virtualization. It uses the validated TVB simulators to generate brain network activity, as well as analysis and visualization tools.

In what follows, we will show how the platform can be used to virtualize not only individual mouse brains (based on dMRI connectome) but also to construct very detailed connectome-based models using tracer data obtained by the Allen Institute for Brain Science (Oh et al., 2014).

As a worked example to show how the platform can be used to generate predictions or interpret data, we will simulate resting state dynamics in a control and “epileptic” mouse and seizure propagation. We will also show how to integrate TVMB in a research project in which theoretical and experimental approaches benefit from one another.

## Materials and Methods

**Figure 1.**
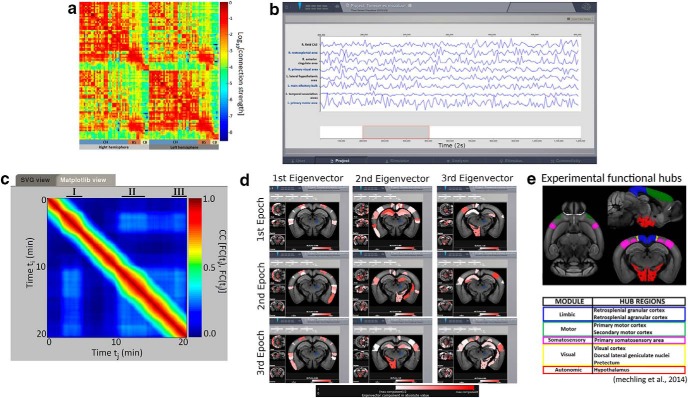
***A***, We used The Allen Connectivity Builder to build the structural connectivity matrix. The color map represents the connections strengths with a base-ten logarithmic scale. The resolution of the grid is 100 μm; the weights of the matrices are defined as the ratio between the projection and the injection density; all the areas in the parcellation have at least one injection experiment that has infected >50 voxels in those areas; the matrix contains only regions with a volume >2 mm^3^. ***B***, Simulated resting state BOLD time series using the connectome built in ***A***, and the eMFM to model the dynamics of each brain area. ***C***, FCD matrix obtained from the time series. The three black segments (I, II, and III) correspond to epochs of stability of the FCD identified with the spectral embedding technique. ***D***, Functional hubs detected *in silico* mapped on brain sections using the brain region volume visualizer. Images in the same row represent the plotting of the eigenvectors components, in absolute value, of the FC belonging to the same epoch. Images organized in different columns refer to eigenvectors belonging to different eigenvalues of the matrices. The scale used allows highlighting only the brain area associated to component of the eigenvector greater than the half of the maximum component. Such scale permits to efficiently visualize the relative difference between eigenvectors. According to our definition (see Materials and Methods), the areas with warm colors are the hub regions of the brain network defined by the FC matrices calculated over the relative epoch; the importance of each hub region is proportional to the corresponding eigenvalue. ***E***, Experimental resting state networks and the corresponding functional hubs detected in Mechling et al. (2014). The Ipython scripts to obtain the results in the figure are Figure 1-1 and Figure 1-2 (Extended data).

**Figure 2.**
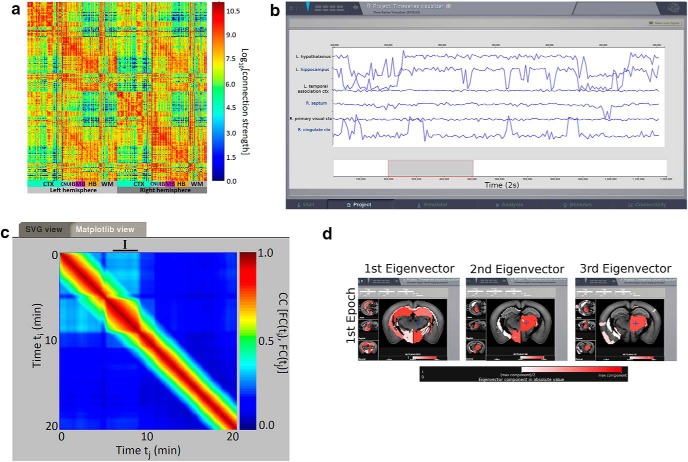
***A***, Connectivity matrix obtained from Calabrese et al. (2015). ***B***, Simulated resting state BOLD time series using the connectome shown in ***A***, and the eMFM to model the dynamics of each brain area. ***C***, FCD matrix obtained from the time series. The black segment identifies the epoch of stability of the FCD identified with the spectral embedding technique. ***D***, Functional hubs detected *in silico* mapped in mouse brain sections using the brain region volume visualizer, as in Figure 1*C*.

**Figure 3.**
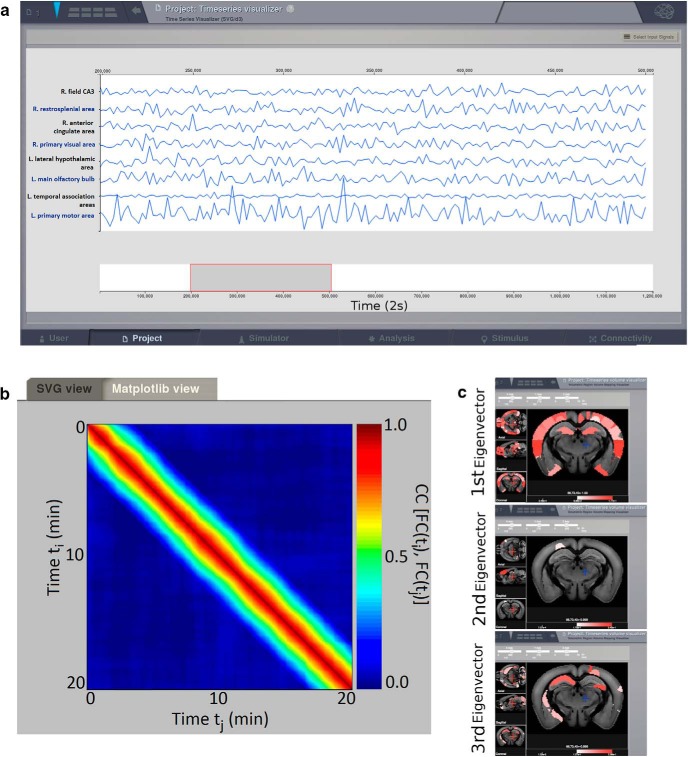
***A***, ***B***, BOLD signals and the corresponding FCD matrix, respectively, obtained by simulating the mouse brain in which some links are removed to mimic epilepsy conditions. ***C***, Functional hubs detected in the epileptic mouse brain after removing links as seen in some forms of epilepsy. The hubs displayed here are extracted from the FC matrix calculated over all the simulated BOLD signals (20 min), i.e., the global FC, since the FCD simulated in the epileptic mouse brain does not present evident sign of nonstationarity and consequently the epoch of stability cannot be detected. The Ipython script to obtain the results in the figure is in Figure 3-1 (Extended data).

**Figure 4.**
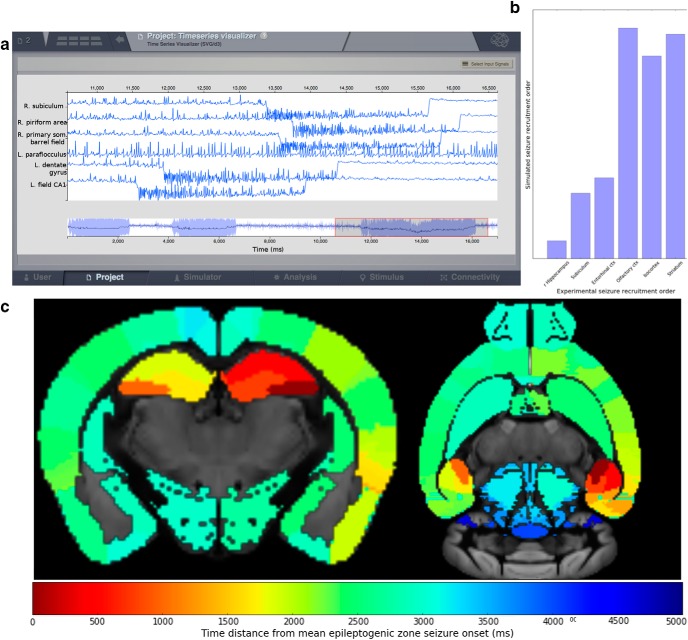
Simulating epileptiform activity in the mouse brain. ***A***, The time series show simulated seizure genesis and propagation (direct current recording) *in silico*. ***B***, The graph shows the propagation pattern. Time 0 corresponds to seizure onset in the left hippocampus. On the *x*-axis, regions are ordered as they are progressively recruited in Toyoda et al. (2013). The *y*-axis shows the average time of recruitment in arbitrary units of these regions after triggering a seizure in the left hippocampus *in silico*. Note the good match between simulated and experimental data. Extensive names of the region composing each group are illustrated in the table in Table 1. ***C***, The time distance from seizure onset in the left hippocampus is given by the color scale and plotted in the brain volume for each region. The Ipython script to obtain the results in the figure is Figure 4-1 (Extended data).

All the methods discussed in what follows are implemented in TVB and freely available to the community. The scripts to reproduce the same results presented in the paper are in Extended data (Figs. 1-1, 1-2, 3-1, 4-1); the tracer-based connectome used here are available in the TVB data folder (…/tvb_data/mouse/).

### The Allen Connectivity Builder

The Allen Connectivity Builder is a pipeline that we have designed to build a complete mouse connectome based on tracer information.

Specifically we define the link between two brain regions according to the anterograde tracing information provided by the Allen Institute of Brain Science and presented in the work of Oh et al. (2014). In the latter, the axonal projections from a given region are mapped by injecting in adult male C57Bl/6J mice the recombinant adeno-associated virus, which expresses the EGFP anterograde tracer. The tracer migration signal is detected with a serial two-photon tomography system. This approach is repeated systematically to collect the information on the tracer migration from several injection sites in the right hemisphere to target regions in both ipsilateral and contralateral hemispheres; for each injection sites several experiments are run and distinct measures are accomplished. The Allen Institute provides its data through an Internet-accessible interface, namely the Allen Software Development kit (Allen SDK), from which TVB, through The Allen Connectivity Builder interface, is able to obtain a volumetric atlas as well as the raw experimental information necessary to build complete mouse brain connectomes. The platform allows to choose the main characteristics of the connectome; specifically the user can set:

1. The resolution of the grid volume in which the data are registered (25, 50, and 100 μm).
2. The definition of the connection strength between source region *i* and target region *j*. Specifically the connection strength can be defined as:
   – The detected projection density (the number of detected pixels in the target region normalized on the total number of pixels belonging to that region).
   – The detected projection energy (the intensity of detected pixels in the target region normalized on the total number of pixels belonging to that region).
   – The ratio between the projection density, defined as explained above, and the injection density of the source region (the number of infected pixels in the source region normalized on the total number of pixels belonging to that region).
  It is possible to choose the characteristics of the brain areas to be included in the parcellation using the two following criteria:
3. Brain areas where at least one injection has infected more than a given threshold of voxels. This kind of selection ensures that only the data with a certain level of experimental relevance is included in the connectome (Oh et al., 2014).
4. Only brain areas that have a volume greater than a given threshold can be included.

The pipeline, once downloaded the raw data from the Allen dataset, cleans the data to obtain a set of experiments in which the injection structures are exactly the same as the target structures and vice versa; this step ensures that the connectome will be a square matrix. Then, the pipeline excludes from the experimental set the area that do not fulfill the criteria set by the user (minimum volume (3) and minimum number of voxels infected (4)).

The experiments of the Allen Institute consider source regions always located in the right hemisphere, thus we build a complete structural connectivity matrix, taking the mirror image of the right hemisphere to build the left one. Therefore, if we divide the SC matrix in four blocks R-R, R-L, L-R, and L-L (clockwise order starting from upper left), we will have the symmetries R-R = L-L and R-L = L-R. This assumption is justified by the fact that the mouse brain shows a high degree of lateral symmetry (Calabrese et al., 2015).

The connection strength between a given region and another one is averaged across all the experiments that use as source and target regions those particular brain areas.

The Allen Connectivity Builder approximates the length of the tracts as the Euclidean distance between the centers of the brain regions; the latter are calculated using the volume built from the Allen SDK.

Finally, The Allen Connectivity Builder creates a region volume mapping, i.e., a 3D matrix which represents the volume of the mouse brain, by modifying the annotation volume downloaded from the Allen SDK. In particular the volume is built so that the entries of the 3D volume matrix range from -1 (background) to N-1, where N is the total number of areas in the connectivity: entries in the volume equal to *i* – 1 label the brain region whose incoming and outgoing connections are organized in the *i*-th row and *i*-th column of the connectivity matrix.

The code named allen_creator.py is located in the folder TVB folder/tvb/adapters/creators/. The code is composed of a function “launch” that calls several functions (all written in the file allen_creator.py) that perform all the actions described above. To work outside TVB, users need to copy and paste all the functions that they need from the allen_creator.py.

The volumes and the connectivities used in the present work have a resolution of 100 μm and each connection strength is defined as the ratio between projection density and injection density. The areas included in the parcellation have a volume >2 mm^3^ and they have >50 voxels infected in at least one injection experiment. The connectome obtained is in the TVB data folder (/TVB_Distribution/tvb_data/lib/python2.7/site-packages/tvb_data/mouse/); the instructions to obtain it through the TVB Jupiter interface are in script Figure 1-1 (Extended data).

## Resting state dynamics

### Brain model

The mean activity of each brain region, composing the mouse brain network, is described by the reduced Wong Wang model (Wong and Wang, 2006). In this approach, the dynamics of a brain region is given by the whole dynamics of excitatory and inhibitory populations of leaky integrate-and-fire neurons interconnected via NMDA synapses. In this work, we take into account this model with a further reduction performed in Deco et al. (2013b): the dynamics of the output synaptic NMDA gating variable *S* of a local brain area *i* is strictly bound to the collective firing rate *H_i_*. The resulting model is given by the following coupled equations:

(1) $$\frac{dS_{i}(t)}{dt}=-\frac{S_{i}}{\tau_{s}}+(1-S_{i})\gamma H_{i}+\sigma \eta_{i}(t)$$

(2) $$H_{i}=\frac{ax_{i}-b}{1-exp(-d(ax_{i}-b))}$$

(3) $$x_{i}=wJ_{N}S_{i}+J_{N}G\sum_{j}C_{ij}S_{j}+I_{0}$$

where *x_i_* is the synaptic input to the *i*-th region. *γ* is a kinetic parameter fixed to 0.641, *τ_s_* is the NMDA decay time constant and its value is 100 ms; *a*, *b*, and *d* are the parameters of the input and output function *H* and are, respectively, equal to 270 *nC*^– 1^, 108 Hz, 0.154 s. *J_N_* = 0.2609 nA is an intensity scale for the synaptic input current. *w* is the local excitatory recurrence and *I*_0_ is the external input current. *G* is the coupling strength, i.e., a scalar parameter which scales all the connection strengths *C_ij_* without altering the global topology of the network.

The value of the local excitatory recurrence, *w*, and the external input current, *I*_0_, are set, respectively, to 0.3 nA and 1 to enrich the nonlinearity of the dynamics of each brain region. Indeed in this case, studying the dynamics of isolated brain areas (*G* = 0 in Eq. 3), it is possible to notice that each brain area is in a bistable state and it oscillates between high and low activity fixed points (Hansen et al., 2015). When the brain areas are connected in the network (i.e., *G* ≠ 0 in Eq. 3), it is not possible to find the analytic solution of $\frac{dS}{dt}$ (Eq. 1), and thus the attractors of the system. The behavior of the connected brain network can be assessed by simulating the system in a deterministic fashion until it reaches the stationary state; repeating this approach several times it is possible to obtain a rich repertoire of stationary states (i.e., attractors of the system) that can be classified through clustering techniques as described previously (Golos et al, 2015). It has been noticed (Hansen et al., 2015) that enriching the nonlinearity of each brain areas has an impact on the global network by introducing attractors that are not in trivial relation with the anatomic connectivity. We refer to this model as the enhanced nonlinearity mean-field model (eMFM). The implementation of the eMFM in a brain network offers the chance to study the nonstationary features of the functional connectivity (FC) patterns.

The values of G, together with the value of the noise amplitude *σ* of the normally distributed stochastic variable *η_i_*, are tuned, respectively, to 0.096 and 5.1 × 10^– 3^. In absence of experimental data, the optimal values of the model parameters cannot be assessed through data fitting techniques; we have choose these model parameters since they allow the system to satisfy the two following conditions. (1) The system is in unstable state so that it is able to explore several brain configurations and it has been shown that this is the optimal range for simulating resting state activity (Deco et al., 2013a). The range of coupling strengths for which the system displays multistability is identified by building its bifurcation diagram as described in Deco et al. (2013b). (2) The system is able to reproduce the checkboard pattern of the FC dynamics (FCD) observed in experimental data; this condition is satisfied for a smallest subset of coupling strength values identified with the first condition (Hansen et al., 2015; Deco et al., 2017). We have use the FCD metrics as clue of goodness of our simulation, since it has been identified in the literature as an important resting state feature (Hutchison et al., 2013; Preti et al., 2016).

### Integration scheme and BOLD signals

Model equations are numerically solved using the Euler integration method with a fixed integration step of 0.1 ms.

Simulated BOLD signal is obtained by converting the simulated synaptic activity (Eq. 1) using the Balloon-Windkessel method (Friston et al., 2000) with the default value implemented in TVB (Sanz-Leon et al., 2015).

The BOLD time series are down-sampled to 2 s and 20 min total length.

### Functional connections

Functional connections in the simulated time series are explored from both spatial and temporal point of views using, respectively, the FC and the FCD.

The *ij*-th element of the FC matrix is calculated as the Pearson correlation between the BOLD signal of the brain region *i* and of the brain region *j*.

To estimate the FCD, the entire BOLD time series is divided in time windows of a fixed length (3 min) and with an overlap of 176 s; the data points within each window centered at the time *t_i_* were used to calculate FC(*t_i_*). The *ij*-th element of the FCD matrix is calculated as the Pearson correlation between the upper triangular part of the *FC*(*t_i_*) matrix arranged as a vector and the upper triangular part of the *FC*(*t_j_*) matrix arranged as a vector.

To observe signal correlations at frequency greater than the typical one of the BOLD signals, the sliding window length is fixed to 3 min, since, as demonstrated by Leonardi and Van De Ville (2015), the nonspurious correlations in the FCD are limited by high-pass filtering of the signals with a cutoff equal to the inverse of the window length.

The FCD matrix allows identifying the epochs of stable FC configurations as blocks of elevated inter-*FC*(*t*) correlation; these blocks are organized around the diagonal of the FCD matrix (Hansen et al., 2015).

### FCD segmentation: spectral embedding

To identify the epochs of stable FC configurations, we used the spectral embedding method, that permits to group together the nodes of the FCD, i.e., the different time windows, in clusters.

The spectral embedding is a general cluster technique founded on the possibility to map the nodes of the network in the Euclidean space such that the Euclidean distance between the nodes in the space corresponds with the distance between the nodes in the network.

To implement this idea, it is necessary to define the notion of distance between nodes in a network; this is made introducing the concept of the commute distance *c_ij_* between the nodes *i* and *j* that is defined as the expected number of steps in a random walk starting to travel from node *i* to node *j*, and back (Von Luxburg, 2007).

To mathematically define *c_ij_* it is necessary to introduce some quantity. Let us consider a graph that has an adjacency matrix *W*, i.e., a matrix whose element *w_ij_* is the weight of the link between node *i* and *j*, that in our case is the FCD matrix; it is possible to define the laplacian of the graph as:

(4) $$L=D-W,\text{ where }D=\left[\begin{array}{ccc} \sum_{j}w_{1,j} & & \\ & \ddots & \\ & & \sum_{j}w_{N,j} \end{array}\right]\;\;.$$

Let us denote with |*e_i_* > the eigenvector *i* of the Laplacian; if the matrix *U* is the matrix whose columns are the eigenvectors of *L*, and Λ the diagonal matrix with the eigenvalues *λ_i_* on the diagonal, thus it is possible to decompose the Laplacian as: *L* = *U*Λ*U^T^*.

The generalized inverse of the Laplacian is defined as $L^{†}=U\Lambda^{†}U^{T}$, where $\Lambda^{†}$ is the diagonal matrix with on the diagonal 1/*λ_i_* when *λ_i_* is different from zero, otherwise zero. Thanks to $L^{†}$ it is possible to express the commute distance between node *i* and *j* as:

(5) $$c_{ij}=vol(V)<e_{i}-e_{j}|L^{†}|e_{i}-e_{j}>\;\;,\text{ with }vol(V)=(\sum_{i}^{N}\sum_{j}^{N}w_{ij})\;\;.$$

The variable |*z_i_* > maps the vertex *v_i_* in the Euclidean space ($z_{i}\in {ℜ}^{N}$) such that the Euclidean distance between node *i* and *j* is equal to the commute distance *c_ij_* of the nodes in the graph if and only if:

(6) $$c_{ij}^{2}=vol(V)||z_{i}-z_{j}|{|}^{2}\;\;\Rightarrow \;\;<e_{i}-e_{j}|U\Lambda^{†}U^{T}|e_{i}-e_{j}>=<z_{i}-z_{j}|z_{i}-z_{j}>\;\;,$$

from which it follows that < *z_i_*| corresponds to the *i*–th row of the matrix $U\sqrt{\Lambda^{†}}$.

### Functional hubs

The FC matrix of each epoch defines a functional network; for each functional network, we identify the hub regions with an approach analogous to the one used in graph theory for defining the eigenvector centrality of a network node (Newman, 2008).

Let us define the functional centrality $\phi^{\left(i\right)}$ of a brain region *i* as the sum of the functional centralities of the neighboring brain regions weighted on the functional connection strength *fc_ij_*:

(7) $$\phi^{\left(i\right)}=\frac{1}{\lambda }\sum_{j=1}^{N}fc_{ij}\phi^{\left(j\right)}$$

x where *λ* is a constant. Defining the vector $\vec{\phi }$ as the column vector whose components are the functional centrality of each network region, we can rewrite the previous equation in matrix form:

(8) $$\overset{\to }{\phi }=\frac{1}{\lambda }FC\overset{\to }{\phi }\;\;.$$

It is simple to notice that $\overset{\to }{\phi }$ is the eigenvector of the FC matrix associated with the eigenvalue *λ*. Since the FC is a real symmetric matrix (thus diagonalizable), we can decompose it as:

(9) $$\begin{array}{ll} FC= & \Phi \Lambda \Phi^{T}=\\ = & \left[\begin{array}{ccc} \overset{\to }{\phi_{1}} & \cdots & \overset{\to }{\phi_{N}} \end{array}\right]\left[\begin{array}{ccc} \lambda_{1} & & \\ & \ddots & \\ & & \lambda_{N} \end{array}\right]\left[\begin{array}{c} {\overset{\to }{\phi_{1}}}^{T}\\ \vdots \\ {\overset{\to }{\phi_{N}}}^{T} \end{array}\right]=\\ = & \overset{N}{\underset{i=1}{\sum }}\lambda_{i}\overset{\to }{\phi_{i}}{\overset{\to }{\phi_{i}}}^{T}\;\;. \end{array}$$

It follows that the magnitude of the eigenvalue gives a measure of the role of the corresponding eigenvector in reproducing the original matrix.

Taking into account all these observations, we identify the functional hub regions of the mouse brain as the regions with the largest eigenvector components, in absolute value, associated with the three largest eigenvalues of the FC matrix.

The script to run resting state simulation with the tracer-based connectome is in Figure 1-2 (Extended data).

### Modeling altered connectomes in pathologic contexts

TVB allows manipulating the structural connectivity by selectively changing the strength of the connections between brain areas to mimic structural lesions. Using this tool, we have simulated mouse brain dynamics mimicking some aspects of the anatomic reorganization found in mesial temporal lobe epilepsy: the neuronal connection lost in hippocampal regions, in particular fields CA1 and CA3 (Esclapez et al., 1999). To reproduce this feature *in silico*, we have removed all the in-coming and out-coming connections of fields CA1 and CA3 of the hippocampus and then scaled all the connection strengths by a constant factor, so that the total weights of the modified SC is equal to the one of the original matrix. We simulated the resting state BOLD activity and we calculated the FCD matrix as described in the previous sections.

The script to run resting state simulation in pathologic condition is in Figure 3-1 (Extended data).

### Epileptic spread *in silico*

#### The epileptic network node model

The Epileptor (Jirsa et al., 2014) is a model describing the onset (through a saddle-node bifurcation), the time course and the offset (through a homoclinic bifurcation) of seizures with five state variables that operate at three different time scales. The variable that guides the neural population through the bifurcations is the slow permittivity variable, *z*, which operates at the slowest time scale. Ensemble 1, comprising the variables *x*_1_ and *y*_1_, describes the fast discharges registered during ictal states and stable state observed during interictal states; it operates at the fastest timescale. Finally, ensemble 2 (*x*_2_, *y*_2_) operates at the intermediate time scale and accounts for spike-and-wave events. The interaction between the variables of the system is the following: ensemble 1, through the function *g*(*x*_1_), excites ensemble 2, which in turn inhibits ensemble 1 through $f_{1}(x_{1},x_{2})$; both the ensembles are coupled to the slow variable, and the first ensemble acts directly on *z*. Proix et al. (2014) propose a permittivity coupling between brain areas via a linear difference coupling function that links the fast subsystems with the slow variable *z* with the weights given by the distance *c_ij_*.

The full model equations read:

$${\overset{˙}{x}}_{1,i}=y_{1,i}-f_{1}(x_{1,i},\;x_{2,i})-z_{i}+I_{i}\;{\overset{˙}{y}}_{1,i}$$

$${\overset{˙}{y}}_{1,i}=1-5(x_{1,i}{)}^{2}-y_{1,i}\;$$

(10) $${\overset{˙}{z}}_{i}\left\{ \begin{array}{c} r(4(x_{1,i}-x_{0,i})-z_{i}-0.1z_{i}^{7})+K_{z}\underset{j}{\sum }c_{ij}(x_{1,i}-x_{1,i})\\ ifz_{i}<0\\ r(4(x_{1,i}-x_{0,i})-z_{i})+K_{z}\underset{j}{\sum }c_{ij}(x_{1,i}-x_{1,i})\\ ifz_{i}<0 \end{array}\right.\;$$

$${\overset{˙}{x}}_{2,i}=-y_{2,i}+x_{2,i}-(x_{2,i}{)}^{3}+I_{2}+0.002\cdot g(x_{1,i})-0.3(z_{i}-3.5)\;$$

$${\overset{˙}{y}}_{2,i}=\frac{1}{\tau }(-y_{2,i}+f_{2}(x_{2,i})),$$

where:

(11) $$f_{1}(x_{1,i},x_{2,i})=\{\begin{array}{ll} 3x_{1,i}^{2}-x_{1,i}^{3} & \text{if }x_{1,i}<0\\ (0.6{\left(z_{i}-4\right)}^{2}-x_{2,i})x_{1,i} & \text{if }x_{1,i}\geq 0 \end{array}$$

(12) $$f_{2}(x_{1,i},x_{2,i})=\{\begin{array}{ll} 0 & \text{if }x_{2,i}<-0.25\\ 6(x_{2,i}+0.25) & \text{if }x_{2,i}\geq -0.25 \end{array}$$

(13) $$g(x_{1,i})=\int_{t_{0}}^{t}e^{-\gamma (t-\tau )}x_{1,i}(\tau )d\tau$$

with $I_{1}=3.1,\;I_{2}=0.45$, *τ* = 10, *γ* = 0.01; the permittivity coupling term *K_s_* is fixed to -60.

The degree of epileptogenicity *x*_0_ of a brain region *i* is a parameter that establishes if the region generates seizures autonomously.

#### Integration scheme and epileptogenicity zone

The epileptogenic zones in the model are nodes of the network that are implemented in the simulation with an epileptogenicity value, *x*_0_, so that, for those nodes, the transition between the preictal and the ictal state occurs spontaneously (Proix et al., 2014). An isolated brain area with *x*_0_ ≤ 2.06 is epileptogenic, otherwise the area is in its equilibrium state and it can generate seizures only if an external stimulus pushes it through the transition and makes it fall in the propagation zone (Proix et al., 2014).

In the simulated mouse brain, the classification of brain areas in epileptogenic and propagation zone follows the experimental results of the work of Toyoda et al. (2013), in which the authors, using recording electrodes, evaluate the seizures propagation in rats with spontaneous seizures. The authors observe that the earliest seizure activity is recorded most frequently within the hippocampal formation and then spreads, in chronological order, in the subiculum, the entorhinal cortex, the olfactory cortex, the neocortex and the striatum; in 7 over 10 rats analyzed in the paper the epileptogenic region is likely identified in either hemisphere. Accordingly we set as epileptogenic, *x*_0_ = –1.9 the left hippocampal regions (field CA1, field CA3, and dentate gyrus), and we set all other regions as propagation zones, *x*_0_ = –2.1.

The differential equations of the model are integrated with the Heun stochastic method with an integration step equal to 0.04 ms; we use additive white Gaussian noise in the fast variables (*x*_2_ and *y*_2_) with mean zero and variance 0.0025. The signals are down-sampled to 1 ms. We set the pre-expression monitor to keep track of the local field potential, defined in the Epileptor as –*x*_1_ + *x*_2_, as well as the slow permittivity value *z*.

We define the time at which seizure initiates in a large brain region, as for example the olfactory cortex, as the mean of the seizure onset time of all the network nodes composing that region (see Table 1); to evaluate the chronological order of areas recruitment we define the seizure onset latency of a region as the difference between the time at which the seizure initiates in that region and the time at which the seizure has started in the epileptogenic zone, i.e., the hippocampal regions.

The script to simulate epileptic activity in mouse brain is in Figure 4-1 (Extended Data).

## Results

### Virtualizing the mouse brain

#### Tracer-based connectome

To exploit present (and future) high-resolution structural information of the Allen Institute, we designed The Allen Connectivity Builder, a pipeline, which uploads their raw data and processes it to create a connectome and its brain volume representation. The user chooses four sets of parameters: the resolution of the tracing data (1); the way the connection strengths are calculated (2); and the criteria used to include or not a given injected region based on its volume (3); and its experimental significance (4). The pipeline then computes automatically the averaged connection strength between any two regions. Since injections were only performed in the right hemisphere and since the mouse brain shows a high degree of lateral symmetry (Calabrese et al., 2015), the pipeline uses the mirror image to build the left hemisphere. If time delays are considered as an important variable to simulate whole-brain activity, the length of each axonal tract becomes a key parameter. The Allen Connectivity Builder approximates the length of the tracts as the Euclidean distance between the centers of two regions. Finally, the pipeline automatically builds the brain volume using the same parcellation as used to build the connectome. TVMB includes a region volume mapping visualizer to display the brain volume as sections and the results of the computations in the brain sections.

An example of a structural connectivity matrix obtained through the Allen connectivity builder is shown in Figure 1*A* and the corresponding volume sections in Figure 1*D*.

10.1523/ENEURO.0111-17.2017.1-1 Figure 1-1 The script shows how to build a tracer-based connectome of mouse brain, using TVMB. Download Figure 1-1, ZIP file.

10.1523/ENEURO.0111-17.2017.f1-2 Figure 1-2 The script shows how to simulate and analyze resting state dynamics in a tracer-based mouse connectome, as described in the paper. Download Figure 1-2, ZIP file.

#### dMRI-based connectome

TVMB can also make use of user-based dMRI data, enabling the virtualization of individual mouse brains. to use the analysis tools and the visualizer above, the brain volume should be uploaded in nifti format with the same parcellation as the connectome.

As an example, we have used here the high-resolution open-source mouse connectome of Calabrese et al. (2015; Fig. 2*A*), which we have embedded in the Allen volume (Fig. 2*D*). In the general case, the user needs to upload the following files: (1) a weight matrix, i.e., a square matrix whose rows and columns label the areas in the parcellation and whose entry (*i*, *j*) represents the values of the connection strength between region *i* and region *j*; (2) a file containing the labels of the brain regions; and (3) the list of Cartesian triplets that specify the spatial location of each region (Sanz-Leon et al., 2015). As exhaustively explained in the TVB documentation (http://docs.thevirtualbrain.org/index.html), it is possible to provide additional information as the lengths of the tracts connecting the brain areas, or a file containing a vector providing a way of distinguishing cortical from subcortical regions, or the volumes where the connectome is embedded in nifti format, etc.

#### Simulated brain activity

Once a virtual brain is constructed, the TVB environment generates a large-scale brain network equation (Jirsa, 2009) offering multiple ways to produce electrophysiological and neuroimaging signals, and analyze their dynamics.

We present three illustrative examples on how mouse brain network simulations can be accomplished. The scripts and the data necessary to reproduce all the simulations and results presented here are in Extended data.

#### Resting state activity in the “healthy” brain

Since recent studies highlighted the importance of studying FCD (Allen et al., 2014, Hansen et al., 2015) and the functional hubs of rodents brain (Mechling et al., 2014; Liska et al., 2015) during resting state activity, we introduce an analyzer able to calculate the FCD and to extract the functional hubs (details of the algorithms in Materials and Methods).

We focus on the nonstationary nature of the fMRI FC in resting state observed both in humans (Chang and Glover, 2010; Allen et al., 2014) and in rodents (Keilholz et al., 2013, Liang et al., 2015). Using the simulator tool of TVB we simulate the resting state activity using the reduced Wong Wang model (Wong and Wang, 2006) in the dynamical regime studied by Hansen et al. (2015). The model differs from previous resting state models (Deco and Jirsa, 2012, Deco et al., 2013b) by having a richer dynamical repertoire for each brain region, which results in a greater number of attractors for the global system.

The BOLD signals and the corresponding FCD matrix are shown in Figure 1*B*,*C*, respectively. The blocks around the diagonal of the FCD matrix correspond to time intervals during which the FC(*t*)s are strongly correlated; following the work of Hansen et al. (2015), we call these periods epochs of stability. The FCD analyzer, using the spectral embedding algorithm, detects three epochs of stability (Fig. 1*C*, black lines) in the FCD matrix. As explained in Materials and Methods, it is possible to identify the central nodes of the *i*-th network (*i* = 1,2,3), i.e., the functional hub regions of the *i*-th epoch, as the nodes linked to the largest components associated with the largest eigenvalues of the FC matrix computed over the *i*-th epoch. The functional hubs identified using this argument by the FCD analyzer are plotted in the mouse brain sections in Figure 1*D*.

It is possible to notice several analogies between the simulated functional hubs and the ones previously reported in literature. In particular, the hypothalamus, the visual and somatosensory cortex have been identified as hubs when analyzing resting state networks in mice (Fig. 1*E*; Mechling et al., 2014). In addition, we find that the agranular insular area is associated to the largest component of the first eigenvector of all epochs (i.e., the most salient hub) in keeping with the experimental data of Liska et al. (2015). Finally, it is interesting to note that the agrunar insular area, the cingulate and temporal cortex are also hub regions in humans (van den Heuvel and Sporns, 2013). Since these hubs are altered in neurologic disorders (Buckner et al., 2009; Crossley et al., 2014), it is straightforward to predict functional consequences after modifying hubs *in silico*. This illustrates a potential use of TVMB.

Brain activity can be simulated also in individual virtual mouse brain built from fMRI diffusion data as explained before. As an example, we uploaded the detailed dMRI connectome from Calabrese et al. (2015) and simulated subsequent resting state activity of its BOLD signals (Fig. 2).

#### Resting state activity in epilepsy

TVMB can be used to assess the functional consequence of the anatomic reorganization that takes place in most, if not all, neurologic disorders. Temporal Lobe Epilepsy is a prototypical example of neurologic disorder with well-described anatomic alterations (Esclapez et al., 1999, Chen and Buckmaster, 2005) and functional reorganizations (Centeno and Carmichael, 2014).

Using the tracer-based connectome described above, we removed the connections from the hippocampal CA3 and CA1 regions known to be lost in some forms of medial temporal lobe epilepsy. The simulated BOLD and the corresponding FCD are shown in Figure 3.

10.1523/ENEURO.0111-17.2017.f3-1 Figure 3-1 The script shows how to simulate and analyze resting state dynamics in an epileptic mouse brain, as described in the paper. Download Figure 3-1, ZIP file.

The comparison between the activity of the healthy and epileptic brain at the level of a single region (Figs. 1*B*, 3*A*, respectively), does not provide any particular insight. However the differences in brain activity between the two conditions are revealed at the network level when computing the FCD (Fig. 3*B*). The functional connections that emerge in the epileptic brain are not correlated in time resulting in a suppression of the switching behavior of the FCD, as compared with the control connectome (Fig. 1*C*). As a result the functional hubs are modified. Since there is no switching, only hubs of global FC can be identified (Fig. 3*C*).

#### Seizure propagation

TVB also contains numerous models to generate EEG-like activity, including the Epileptor to simulate seizure genesis and propagation (Jirsa et al., 2014, Proix et al., 2014).

As an experimental reference, we used the electrophysiological recordings performed by Toyoda et al. (2013) in a rat model of temporal lobe epilepsy. Based on the latter results, we used the left hippocampal regions as epileptogenic zones, and analyzed how and where seizures propagated *in silico*.

The results of the simulation are shown in Figure 4. Each region is characterized by a specific time of seizure onset. The chronological order of the different areas recruited during seizure propagation is shown in Figure 4*B*,*C*. The brain areas in abscissa in Figure 4*B*are sorted according to the seizure onset latency rank found by Toyoda et al. (2013) in rats. Despite the difference in species (rat versus mouse), there is a remarkable analogy with experimental results, suggesting that the structural connectome (and the time delays it imposes) plays a key role in the spatiotemporal pattern of seizure propagation as already reported in humans (Jirsa et al., 2017, Proix et al., 2017).

10.1523/ENEURO.0111-17.2017.4-1 Figure 4-1 The script shows how to simulate epileptic seizure propagation in mouse brain, as described in the paper. Download Figure 4-1, ZIP file.

**Table 1.** List of regions used for the simulation.

Group	Components
Hippocampus	Field CA1
	Field CA3
	Dentate gyrus
Subiculum	Subiculum
Entorhinal cortex	Entorhinal area, lateral part
	Entorhinal area, medial part, dorsal zone
Olfactory cortex	Main olfactory bulb
	Anterior olfactory nucleus
	Piriform area
Neocortex	Primary motor area
	Secondary motor area
	Primary somatosensory area, nose
	Primary somatosensory area, barrel field
	Primary somatosensory area, mouth
	Primary somatosensory area, upper limb
	Supplemental somatosensory area
	Gustatory areas
	Dorsal auditory area
	Primary auditory area
	Ventral auditory area
	Primary visual area
	Anterior cingulate area, dorsal part
	Anterior cingulate area, ventral part
	Agranular insular area, dorsal part
	Retrosplenial area, dorsal part
	Retrosplenial area, ventral part
	Temporal association areas
	Right perirhinal area
	Ectorhinal area
Striatum	Caudoputamen
	Nucleus accumbens

In order to compare the simulated spread of the seizure activity with the empirical one recorded by Toyoda et al. (2013), we group together brain areas located in proximity to the position of the electrodes used by the authors.

### Interpreting and planning experiments with TVMB

#### Interpreting experimental data with TVMB

**Figure 5.**
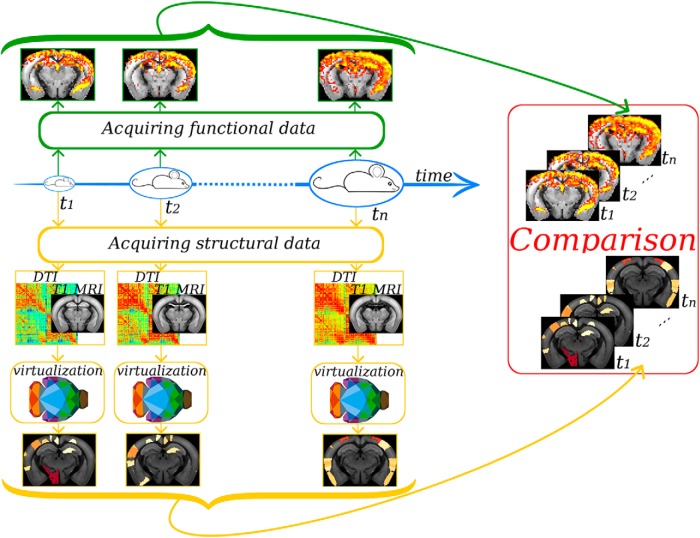
The cartoon illustrates how it is possible to use TVMB to do predictions when studying aging. A mouse can be scanned at different times *t* extracting anatomic and functional brain information. The anatomic information can be processed to obtain a connectome that can be used in TVMB to create a virtual mouse at each time step. The functional experimental information can be compared with the predictions done in TVMB, investigating how, for example, anatomic modifications during aging affect whole-brain dynamics. Multiple other testable predictions can be done. For example, explore *in silico* which types of neurones can be stimulated (or silenced) to activate specific resting state networks. The predictions can then be tested in ad hoc transgenic mice with optogenetics.

Physiologic (e.g., normal aging) and pathologic processes (e.g., neurologic disorders) are associated with both structural (connectome) and functional (resting state networks) alterations. A central issue in neuroscience research is to understand how much structural alterations can account for functional ones. At present, both observations remain at the correlation level. In the case of aging, DTI and rsfMRI can be obtained at different times in a given animal (Figure 5). A virtual brain can be constructed at each time step, to simulate whole-brain dynamics. Following data fitting, alterations found specifically at time *t* + 1 experimentally can be introduced in the connectome measured at time *t*. If the resulting *in silico* rsfMRI reproduces that experimentally measured at time *t* + 1, it is possible to propose that these structural alterations are sufficient to explain the changes in whole-brain dynamics.

#### Planning experiments with TVMB

We present two of the many possibilities offered by the platform. Brain surgery and stimulation are two common procedures used to treat patients, e.g., for epilepsy and Parkinson’s disease. After virtualizing a mouse model of these pathologies at a specific stage of their evolution, researchers can perform neurosurgery *in silico* and predict the efficacy of the procedure. Likewise, *in silico* stimulation of brain regions is straightforward in TVMB, which allows studying how resting state dynamics can be manipulated (Spiegler et al., 2016). The predictions thus generated can then be tested experimentally *in vivo* in the same mouse that was used to make them. Novel preclinical strategies may thus be tested in mice, before their possible clinical transfer.

Many brain functions require dynamical interactions and information transfer between numerous brain regions. The contribution of a given region is thus difficult to evaluate a priori. Using a parameteric study in TVMB, it is possible to predict which regions play a key role by successively activating and inactivating them. Then, one can plan the experiment, choosing the appropriate transgenic mouse line to control the identified region with optogenetics or pharmacogenetics. Such a priori knowledge provided by the *in silico* approach would considerably accelerate research.

## Discussion

TVMB opens a new set of research possibilities: it allows researchers, from different fields, to easily build specific/individual mouse brains (using various resolutions, weighting definitions and parcellations), to simulate different dynamical behaviors (using diverse neural population models, numerical integration schemes, and simulated neuroimaging modalities) and finally to analyze the results.

However, while TVMB is a highly generic framework, its underlying mathematical framework and simulation techniques make standard assumptions, among which the two most essential are that (1) the average activity of large populations of neurons is a meaningful quantification of the phenomena to be modeled and (2) the statistics of white matter fibers sufficiently describes how regions interact. Both resting state dynamics and seizure propagation, as demonstrated above, satisfy these assumptions. On the other hand, for example, fine grained spike timing effects would not well described within TVMB’s mathematical framework.

The wide range of possibility offered by rodent experiments will easily accommodate the validation of the parameterization required by all the modeling approaches contained in the software. This validation sometimes can proceed at a qualitative level for phenomenological models, such as the Kuramoto model of synchronization, but many detailed biophysical models allow for quantitive comparison with empirical data, such as spike timing (Brette and Gerstner, 2005). At the whole-brain level, TVMB allows for direct comparison with common modalities such as EEG, MEG, and fMRI, or common statistics thereupon such as FC; these comparisons allow for the characterization of parameter values in terms of their fit with empirical data and thus biological validity. The experimentally observed functional characteristics of the mouse brain (e.g., a functional hub during resting state or the effects of specific connections removal) can be easily imposed in the output of the virtual system, and through data fitting algorithms, it will be possible to retrieve the parameters of the model that give rise to that particular functional behavior. In this way, closing the circle, the reliability of the new predictions accomplished with the fitted parameter set will be improved; additionally the knowledge of the key features responsible of the different functional behavior allows to control and manipulate the system *in silico*, and, going a step further, also *in vivo*.

TVMB thus offers not only a conceptual framework to interpret neuroimaging data but, combined with experimental approaches, it also offers an operative framework to investigate the causal links between structure and function in the brain.

It is important to note that, to build the Allen connectome, we took the mirror image of the right hemisphere to build the left one, since injections of tracers were performed in the right hemisphere only (Oh et al., 2014). However, contrary to humans (Toga and Thompson, 2003), there is a high degree of similarity in terms of connections between the right and the left hemispheres in rodents (Calabrese et al., 2015).

The detailed tracer connectome, that can be built with TVMB, can be used to build a precise brain model, and serve as a reference or template for dMRI-based modeling, since dMRI data suffer from three major limitations, that are: (1) our ignorance about the directionality of the connections, (2) the indirect nature of the measures of connectivity based on water diffusion in white matter, and (3) the fact that complex fiber pathways (as crossing fibers, sharp change in directionality and long brain wirings) cannot be properly detected. How such limitations affect the simulations is not yet established. On the other hand tracer-based connectome are built by averaging experiments over many mice, and the definition of the seed region is based on stereotaxic coordinate rather than on anatomy. A comparison of the effects in simulating brain network using one or the other kind of connectome is needed especially since the only data available in human are dMRI data.

TVMB is an actively developed software, with new versions released regularly with new features. Among those targeted specifically for the mouse, the module which builds connectivities from the public Allen data will continue to evolve as the available dataset becomes richer. For example, when cortical layer annotations become available, it will be possible to construct mouse connectivities in which the cortical layers are distinct, allowing for example manipulations of inter-layer interactions. As dMRI protocols and tractography techniques become more established for rodent datasets, TVMB has potential to include modules which automate, with visual inspection across each step, the generation of connectomes for individual rodent data, directly from the DICOM slices provided by the acquisition equipment.

## Synthesis

Reviewing Editor: Gustavo Deco, Universitat Pompeu Fabra Decisions are customarily a result of the Reviewing Editor and the peer reviewers coming together and discussing their recommendations until a consensus is reached. When revisions are invited, a fact-based synthesis statement explaining their decision and outlining what is needed to prepare a revision will be listed below. The following reviewer(s) agreed to reveal their identity: William Lytton, Adrián Ponce-Alvarez

A useful paper that introduces the mouse Virtual Brain (TMVB), a mouse version of the human VB which has the advantage of having a connectome based on tracings rather than imaging. Overall a good paper; I have only a few points about possible additional discussion or clarification that might be useful.

This is primarily a methods paper but also features some interesting results. One results is hidden under “It is possible to notice several analogies ...” This could be elaborated on, perhaps with the addition of larger clearer figures with some arrows and specific comparisons (rather than analogies). Fig 2 then shows evaluation of an individual mouse from fMRI data. In recent papers, the group has utilized VB to look at individual patients. Perhaps Fig 2 could be expanded to show more (>= 2) mice if such data is available in order to show that there are detectable differences. Fig 3 and 4 are interesting in that they shows a seizure created by ablating a particular pathway - presumably the comparison of this to the experimental mouse will be saved for another paper.

Python code should be presented textually (and ideally cut&pasteably) as appendices rather than via screenshots as figures.

Is the Allen Connectivity Builder pipeline available (or can it be made available) independent of its use in MVB? -- ie in some standard (or to be made standard) format so can also be used in other simulators? Clarify please.

Discussion points: Is assuming mirror imaging of connectivity an adequate approximation in the mouse? How about in the human? How about between humans -- I would presume that this would specifically not be adequate for the epileptic person or mouse?

Minor:

1. “neuron models are reduced to point neurons” -- can cite specific packages eg BRIAN, NEST, NENGO

2. “available in the TVB data folder.” -- provide a pointer to this specific model; I searched around a bit and couldn't find it.

3. Linux case; Mac case ... -- suggest putting such tech details either into an appendix or better yet into the README for the package. Presumably “does not yet contain” will be temporary so that this will not be needed in the future.

4. Fig 1-2 appears to be an independent result figure and should be elevated to the results sequence if seems worthwhile -- I would suggest that the main sequence of Fig 1-5 is already sufficient and that the hyphenated fig series should all be given as code appendix as above.

-----

Rev. 2:

In the present manuscript, the authors show an extension of The Virtual Brain (TVB), a platform for simulating large-scale models based on human connectomes, to whole brain mouse data. The platform can handle connectome data from both MRI diffusion imaging and fiber-tracing data from the Allen Institute. This is important since in this way model predictions can be tested on transgenic mice that can be manipulated pharmacologically or through optogenetics and recorded during maturation. The new Virtual Mouse Brain is thus a promising tool that could be used to plan theoretically driven experiments and get insights into general principles of brain dynamics.

The method is timely since connectivity data and optogenitic protocols are expected to become more and more accurate. I enjoyed reading the article, the work is clearly written and the methods are clearly presented. However, I have some few concerns that should be addressed by the authors:

1. The authors used a mean field model (eMFM) to simulate the activity of each brain region. The dynamics of the network should be described in more details: how many attractors are accessible to the global system?

2. It is not clear in text why the functional connections (FC) and their dynamics (FCD) would be the best candidate statistics to learn the model parameters (in particular given that FC and FCD are not independent, as shown by equations 7-9), rather than, for example, FC and signal variances or other statistics.

3. The authors claim that the connectome statistics sufficiently determine how regions interact, but note that short term synaptic plasticity is likely to contribute to FCD.

4. I think the manuscript lacks a discussion on which are the advantages/disavantages of using tracer-based connectomes with respect to diffusion imaging datasets. For example, the authors could comment on the effects of using directed connectomes (given by tracer studies) against the undirected ones obtained using diffusion imaging, as directionality significantly affects the network dynamics.

5. Does the hemodynamic model use the firing rates or the synaptic activity?

I hope the authors find these comments helpful.

## References

[B1] Allen EA, Damaraju E, Plis SM, Erhardt EB, Eichele T, Calhoun VD (2014) Tracking whole-brain connectivity dynamics in the resting state. Cereb Cortex 24:663-676. 10.1093/cercor/bhs352 PMC3920766 23146964

[B2] Brette R, Gerstner W (2005) Adaptive exponential integrate-and-fire model as an effective description of neuronal activity. J Neurophysiol 94:3637–3642. 10.1152/jn.00686.2005 16014787

[B3] Buckner RL, Sepulcre J, Talukdar T, Krienen FM, Liu H, Hedden T, Andrews-Hanna JR, Sperling RA, Johnson KA (2009) Cortical hubs revealed by intrinsic functional connectivity: mapping, assessment of stability, and relation to alzheimer’s disease. J Neurosci 29:1860–1873. 19211893 10.1523/JNEUROSCI.5062-08.2009 PMC2750039

[B4] Calabrese E, Badea A, Cofer G, Qi Y, Johnson GA (2015) A diffusion MRI tractography connectome of the mouse brain and comparison with neuronal tracer data. Cereb Cortex 25:4628-4637. 10.1093/cercor/bhv121 PMC4715247 26048951

[B5] Centeno M, Carmichael DW (2014) Network connectivity in epilepsy: resting state fMRI and eeg–fMRI contributions. Front Neurol 5:93. 10.3389/fneur.2014.00093 25071695 PMC4081640

[B6] Chang C, Glover GH (2010) Time–frequency dynamics of resting-state brain connectivity measured with fMRI. Neuroimage 50:81–98. 10.1016/j.neuroimage.2009.12.011 20006716 PMC2827259

[B7] Chen S, Buckmaster PS (2005) Stereological analysis of forebrain regions in kainate-treated epileptic rats. Brain Res 1057:141–152. 10.1016/j.brainres.2005.07.058 16122711

[B8] Crossley NA, Mechelli A, Scott J, Carletti F, Fox PT, McGuire P, Bullmore ET (2014) The hubs of the human connectome are generally implicated in the anatomy of brain disorders. Brain 137:2382–2395. 10.1093/brain/awu132 25057133 PMC4107735

[B9] Deco G, Jirsa VK (2012) Ongoing cortical activity at rest: criticality, multistability, and ghost attractors. J Neurosci 32:3366–3375. 10.1523/JNEUROSCI.2523-11.2012 22399758 PMC6621046

[B10] Deco G, Jirsa VK, McIntosh AR (2013a) Resting brains never rest: computational insights into potential cognitive architectures. Trends Neurosci 36:268–274. 10.1016/j.tins.2013.03.001 23561718

[B11] Deco G, Ponce-Alvarez A, Mantini D, Romani GL, Hagmann P, Corbetta M (2013b) Resting-state functional connectivity emerges from structurally and dynamically shaped slow linear fluctuations. J Neurosci 33:11239–11252. 23825427 10.1523/JNEUROSCI.1091-13.2013 PMC3718368

[B12] Deco G, Kringelbach ML, Jirsa V, Ritter P (2017) The dynamics of resting fluctuations in the brain: metastability and its dynamical cortical core. Sci Rep 7:3095. 10.1038/s41598-017-03073-5 PMC5465179 28596608

[B13] Diesmann M, Gewaltig M-O (2001) NEST: an environment for neural systems simulations. Forschung und wisschenschaftliches Rechnen, Beiträge zum Heinz-Billing-Preis 2001 58:43–70.

[B14] Eliasmith C, Stewart TC, Choo X, Bekolay T, DeWolf T, Tang Y, Rasmussen D (2012) A large-scale model of the functioning brain. Science. Vol. 338 no. 6111 pp. 1202–1205. 10.1126/science.1225266 23197532

[B15] Esclapez M, Hirsch JC, Ben-Ari Y, Bernard C (1999) Newly formed excitatory pathways provide a substrate for hyperexcitability in experimental temporal lobe epilepsy. J Comp Neur 408:449–460. 10.1002/(SICI)1096-9861(19990614)408:4<449::AID-CNE1>3.0.CO;2-R 10340497

[B16] Friston KJ, Mechelli A, Turner R, Price CJ (2000) Nonlinear responses in fMRI: the balloon model, volterra kernels, and other hemodynamics. Neuroimage 12:466–477. 10.1006/nimg.2000.0630 10988040

[B17] Golos M, Jirsa V, Daucé E (2015) Multistability in large scale models of brain activity. PLoS Comput Biol 11:e1004644. 10.1371/journal.pcbi.1004644 26709852 PMC4692486

[B18] Goodman DF, Brette R (2009) The Brian simulator. Front Neurosci 3:26. 10.3389/neuro.01.026.2009 PMC2751620 20011141

[B19] Hansen EC, Battaglia D, Spiegler A, Deco G, Jirsa VK (2015) Functional connectivity dynamics: modeling the switching behavior of the resting state. Neuroimage 105:525–535. 10.1016/j.neuroimage.2014.11.001 25462790

[B20] Hines ML, Carnevale NT (1997) The neuron simulation environment. Neural Comput 9:1179–1209. 9248061 10.1162/neco.1997.9.6.1179

[B21] Hutchison RM, Womelsdorf T, Allen EA, Bandettini PA, Calhoun VD, Corbetta M, Della Penna S, Duyn JH, Glover GH, Gonzalez-Castillo J, Handwerker DA, Keilholz S, Kiviniemi V, Leopold DA, de Pasquale F, Sporns O, Walter M, Chang C (2013) Dynamic functional connectivity: promise, issues, and interpretations. Neuroimage 80:360–378. 10.1016/j.neuroimage.2013.05.079 23707587 PMC3807588

[B22] Izhikevich EM (2003) Simple model of spiking neurons. IEEE Trans Neural Netw 14:1569–1572. 10.1109/TNN.2003.820440 18244602

[B23] Jirsa VK (2009) Neural field dynamics with local and global connectivity and time delay. Philos Trans A Math Phys Eng Sci 367:1131–1143. 19218155 10.1098/rsta.2008.0260

[B24] Jirsa VK, Stacey WC, Quilichini PP, Ivanov AI, Bernard C (2014) On the nature of seizure dynamics. Brain 137:2210–2230. 10.1093/brain/awu133 24919973 PMC4107736

[B25] Jirsa V, Proix T, Perdikis D, Woodman M, Wang H, Gonzalez-Martinez J, Bernard C, Bénar C, Guye M, Chauvel P, Bartolomei F (2017) The virtual epileptic patient: individualized whole-brain models of epilepsy spread. Neuroimage 145:377-388. 10.1016/j.neuroimage.2016.04.049 27477535

[B26] Keilholz SD, Magnuson ME, Pan W-J, Willis M, Thompson GJ (2013) Dynamic properties of functional connectivity in the rodent. Brain Connect 3:31–40. 10.1089/brain.2012.0115 23106103 PMC3621313

[B27] Leonardi N, Van De Ville D (2015) On spurious and real fluctuations of dynamic functional connectivity during rest. Neuroimage 104:430–436. 10.1016/j.neuroimage.2014.09.007 25234118

[B28] Liang Z, Liu X, Zhang N (2015) Dynamic resting state functional connectivity in awake and anesthetized rodents. Neuroimage 104:89–99. 10.1016/j.neuroimage.2014.10.013 25315787 PMC4252714

[B29] Liska A, Galbusera A, Schwarz AJ, Gozzi A (2015) Functional connectivity hubs of the mouse brain. Neuroimage 115:281–291. 10.1016/j.neuroimage.2015.04.033 25913701

[B30] Markram H (2012) The human brain project. Sci Am 306:50–55. 22649994 10.1038/scientificamerican0612-50

[B31] Mechling AE, Hübner NS, Lee H-L, Hennig J, von Elverfeldt D, Harsan L-A (2014) Fine-grained mapping of mouse brain functional connectivity with resting-state fMRI. Neuroimage 96:203–215. 10.1016/j.neuroimage.2014.03.078 24718287

[B32] Newman ME (2008) The mathematics of networks. The New Palgrave Encyclopedia of Economics 2:1–12.

[B33] Oh SW, Harris JA, Ng L, Winslow B, Cain N, Mihalas S, Wang Q, Lau C, Kuan L, Henry AM, Mortrud MT, Ouellette B, Nguyen TN, Sorensen SA, Slaughterbeck CR, Wakeman W, Li Y, Feng D, Ho A, Nicholas E, et al. (2014) A mesoscale connectome of the mouse brain. Nature 508:207–214. 10.1038/nature13186 24695228 PMC5102064

[B34] Preti MG, Bolton TA, Van De Ville D (2016) The dynamic functional connectome: state-of-the-art and perspectives. Neuroimage. Advance online publication. Retrieved Dec 26, 2016. doi: 10.1016/j.neuroimage.2016.12.061. 10.1016/j.neuroimage.2016.12.061 28034766

[B35] Proix T, Bartolomei F, Chauvel P, Bernard C, Jirsa VK (2014) Permittivity coupling across brain regions determines seizure recruitment in partial epilepsy. J Neurosci 34:15009–15021. 10.1523/JNEUROSCI.1570-14.2014 25378166 PMC6608363

[B36] Proix T, Bartolomei F, Guye M, Jirsa VK (2017) Individual brain structure and modelling predict seizure propagation. Brain 140:641-654. 10.1093/brain/awx004 PMC5837328 28364550

[B37] Sanz Leon P, Knock SA, Woodman MM, Domide L, Mersmann J, McIntosh AR, Jirsa V (2013) The virtual brain: a simulator of primate brain network dynamics Front Neuroinform 7:10. 10.3389/fninf.2013.00010 PMC3678125 23781198

[B38] Sanz-Leon P, Knock SA, Spiegler A, Jirsa VK (2015) Mathematical framework for large-scale brain network modeling in the virtual brain. Neuroimage 111:385–430. 10.1016/j.neuroimage.2015.01.002 25592995

[B39] Sinha N, Dauwels J, Kaiser M, Cash SS, Westover MB, Wang Y, Taylor PN (2017) Predicting neurosurgical outcomes in focal epilepsy patients using computational modelling. Brain 140:319-332. 10.1093/brain/aww299 PMC5278304 28011454

[B40] Spiegler A, Hansen ECA, Bernard C, McIntosh AR, Jirsa VK (2016) Selective activation of resting-state networks following focal stimulation in a connectome-based network model of the human brain. eNeuro 3 10.1523/ENEURO.0068-16.2016 PMC5052665 27752540

[B41] Stafford JM, Jarrett BR, Miranda-Dominguez O, Mills BD, Cain N, Mihalas S, Lahvis GP, Lattal KM, Mitchell SH, David SV, Fryer JD, Nigg JT, Fair DA (2014) Large-scale topology and the default mode network in the mouse connectome. Proc Natl Acad Sci USA 111:18745–18750. 25512496 10.1073/pnas.1404346111 PMC4284535

[B42] Toga AW, Thompson PM (2003) Mapping brain asymmetry. Nat Rev Neurosci 4:37–48. 10.1038/nrn1009 12511860

[B43] Toyoda I, Bower MR, Leyva F, Buckmaster PS (2013) Early activation of ventral hippocampus and subiculum during spontaneous seizures in a rat model of temporal lobe epilepsy. J Neurosci 33:11100–11115. 10.1523/JNEUROSCI.0472-13.2013 23825415 PMC3718374

[B44] van den Heuvel MP, Sporns O (2013) Network hubs in the human brain. Trends Cogn Sci 17:683–696. 10.1016/j.tics.2013.09.012 24231140

[B45] Von Luxburg U (2007) A tutorial on spectral clustering. Stat Comput 17:395–416. 10.1007/s11222-007-9033-z

[B46] Wong K-F, Wang X-J (2006) A recurrent network mechanism of time integration in perceptual decisions. J Neurosci 26:1314–1328. 10.1523/JNEUROSCI.3733-05.2006 16436619 PMC6674568

